# Internet use and decision making in community-based older adults

**DOI:** 10.3389/fpsyg.2013.00605

**Published:** 2013-09-17

**Authors:** Bryan D. James, Patricia A. Boyle, Lei Yu, David A. Bennett

**Affiliations:** ^1^Department of Internal Medicine, Rush Alzheimer's Disease Center, Rush University Medical CenterChicago, IL, USA; ^2^Department of Behavioral Sciences, Rush Alzheimer's Disease Center, Rush University Medical CenterChicago, IL, USA; ^3^Department of Neurological Sciences, Rush Alzheimer's Disease Center, Rush University Medical CenterChicago, IL, USA

**Keywords:** internet, decision making, older adults, aging, cohort study

## Abstract

Use of the internet may provide tools and resources for better decision making, yet little is known about the association of internet use with decision making in older persons. We examined this relationship in 661 community-dwelling older persons without dementia from the Rush Memory and Aging Project, an ongoing longitudinal study of aging. Participants were asked to report if they had access to the internet and how frequently they used the internet and email. A 12-item instrument was used to assess financial and healthcare decision making using materials designed to approximate those used in real world settings. Items were summed to yield a total decision making score. Associations were tested via linear regression models adjusted for age, sex, race, education, and a measure of global cognitive function. Secondary models further adjusted for income, depression, loneliness, social networks, social support, chronic medical conditions, instrumental activities of daily living (IADLs), life space size, and health and financial literacy. Interaction terms were used to test for effect modification. Almost 70% of participants had access to the internet, and of those with access, 55% used the internet at least several times a week. Higher frequency of internet use was associated with better financial and healthcare decision making (β = 0.11, *p* = 0.002). The association persisted in a fully adjusted model (β = 0.08, *p* = 0.024). Interaction models indicated that higher frequency of internet use attenuated the relationships of older age, poorer cognitive function, and lower levels of health and financial literacy with poorer healthcare and financial decision making. These findings indicate that internet use is associated with better health and financial decision making in older persons. Future research is required to understand whether promoting the use of the internet can produce improvements in healthcare and financial decision making.

## Introduction

Some of the most complex and influential decisions of modern society are encountered in the later years of life. Older adults face many crucial financial decisions such as maximizing retirement savings, intergenerational transfer of resources, and distinguishing legitimate investment opportunities from scams. Older adults also face major medical decisions such as selecting and negotiating with health plans and providers as well as enrolling government-sponsored service plans. In the increasingly digital world that we live in, the internet has become the primary source of information to aid in decision making in the domains of personal finance and healthcare, trumping even advice from family and friends (Fleishman-Hillard International Communcations and Harris Interactive, 2012). Over time, more and more of the information and services that are helpful—or even necessary—for making these major life decisions are being moved online with the expectation that individuals are functional internet users (Freese et al., 2006). However, many older adults express lack of interest (Morris et al., 2007; Keenan, 2009) or anxiety (Dyck and Smither, 1992) about using computers or the internet, and the aging process can impose obstacles to computer use such as declines in cognition, vision, hearing, and motor skills (Freese et al., 2006; Wagner et al., 2010), resulting in a “Digital Divide” between young and old (Loges and Jung, 2001; Morris et al., 2007). Thus, a large portion of older persons may be cut-off from the online resources that can aid in decisions about their finances and health—while ironically being at a stage in life where these decisions are most influential.

While Americans over age 65 are the age group least likely to use the internet, half of American seniors are now online and they are the fastest growing group of internet users (Zickuhr and Madden, 2012). Older adults who bridge the Digital Divide have been shown to experience a number of positive outcomes compared to those who are not online, including a higher sense of empowerment (Mcmellon and Schiffman, 2002), self-efficacy (Karavidas et al., 2005), and well-being (Chen and Persson, 2002; Shapira et al., 2007), though there have been mixed findings (Dickinson and Gregor, 2006). Despite this encouraging line of research regarding the benefits of internet use in the elderly, there has been little data directly linking internet use to better decision making in older adults. Given the aging of the U.S. population, the economic recession, and the increasing costs of healthcare in America, improving decision making in these domains could have important implications for the quality of life and independence of seniors (Boyle et al., 2013).

The current study is part of an overall program of research aimed to understand the correlates and consequences of impaired decision making in old age; our conceptual framework is guided by existing literature (Finucane et al., 2005) and views decision making as a complex function of multiple resources and abilities, as well as the decision maker's potential to access and utilize those resources and abilities. We have previously shown that cognitive ability (Boyle et al., 2012b) and personality styles (Boyle et al., 2012a) are related to decision making; further, we recently showed that adequate health and financial literacy—the ability to understand and utilize basic information and concepts in these domains—is related to better decision making, over and above the effect of cognitive abilities (James et al., 2012). However, optimal decision making requires more than basic domain-specific literacy; for complex decisions such as choosing mutual funds and health maintenance organization (HMO) plans, access to additional information regarding the benefits and drawbacks of options available is often needed. Thus, in keeping with our conceptual framework, we hypothesized that internet use represents an important vehicle by which older persons may access and utilize the domain-specific resources that are required to successfully comprehend and make optimal choices regarding important life decisions. In support of this idea, more and more Americans report that the internet has improved the way they access information regarding healthcare and personal finances (Howard et al., 2001). However, we are not aware of prior studies that have examined the association of internet use and decision making.

We therefore used data from a cohort of almost 700 non-demented older adults around the greater Chicago area to examine access to and frequency of internet use in persons who were an average of 80 years of age. We tested the hypothesis that more frequent use of the internet in the elderly is related to better decision making ability based on a measure designed to closely approximate decisions made by older persons in the real world in two domains of great salience to older adults: personal finance and healthcare. Alternatively, because persons who are more frequently online may be gathering information from their social contacts, we tested the secondary hypothesis that more frequent email use is related to better financial and healthcare decision making in order to indirectly assess whether expanded connection to social resources could account for the association between internet use and decision making. Although findings supporting these hypotheses using observational data do not provide direct evidence that increasing internet access is causally related to improved decision making in older persons, it is a first step in establishing a link between the internet and decision making in the elderly in the absence of data. Findings from this study could then inform an experiment utilizing an intervention to increase internet use. In controlled analyses, we determined the influences of potential confounders that could induce a spurious association including demographics, cognitive, and physical functioning, socioeconomic status (SES), a variety of psychosocial factors, and literacy regarding basic health and financial concepts. We also tested whether these factors modified the relationship between internet use and decision making ability in interaction models.

## Materials and methods

### Participants

Data for this study came from the Rush Memory and Aging Project, an ongoing longitudinal cohort study of chronic conditions of aging (Bennett et al., 2005). Participants were recruited from around 40 retirement and subsidized housing facilities around the Chicago metropolitan area. All participants agree to annual clinical evaluation conducted by examiners blinded to previous data, included medical history, neurological, and neuropsychological examinations (Bennett et al., 2005). The Memory and Aging Project began in 1997, and enrollment is ongoing. The study was approved by the institutional review board of Rush University Medical Center.

Because the ability of persons with dementia to validly self-report is uncertain, we removed persons diagnosed with dementia prior to the decision making assessment from the analysis. At each evaluation, clinical diagnoses of dementia were conducted using a three stage process including computer scoring of cognitive tests, clinical judgment by an experienced neuropsychologist, and diagnostic classification by an experienced clinician (Bennett et al., 2005). Diagnosis of dementia and probable AD followed the criteria of the joint working group of the National Institute of Neurologic and Communicative Disorders and Stroke and the Alzheimer's Disease and Related Disorders Association (McKhann et al., 1984).

The decision making assessment battery was added to the Memory and Aging Project in 2010. At the time of this analysis, 1676 participants had completed the Memory and Aging Project baseline evaluation. Of those, 531 died and 85 refused further participation before completing the decision making assessment battery, and 112 were deemed ineligible for a first decision making assessment due to significant cognitive, vision, hearing, or language impairment. Of the remaining 860 potentially eligible persons, 728 completed the decision making assessment, 74 had not yet completed their decision making baseline evaluation, and 58 refused the decision making assessment. Of the 728 participants who had completed the decision making assessment, 38 were diagnosed with dementia and were not included in this analysis, leaving 690 eligible persons. Twenty nine of these eligible participants had missing data on the main decision making outcome measure, leaving 661 persons in this analysis. The analytic cohort had a mean age of 82.2 (range: 60–101), a mean of 15.2 years of education, was 76% female, and 91% white, non-Hispanic. Compared to older persons with normal cognition in a comparable population-based cohort of older adults, the Aging, Demographics, and Memory Study (ADAMS), the participants in this study were more highly educated (ADAMS mean = 12.4), more likely to be female (ADAMS frequency: 61%), and slightly more likely to be white non-Hispanic (ADAMS frequency = 89%; Plassman et al., 2008).

### Internet use

As part of the decision making assessment instrument, participants were asked a series of questions regarding technology use, including whether they had access to a computer with internet service (yes or no), and if so, how often they used or searched the internet in the past year (Every day or almost every day, Several times a week, Several times a month, Several times a year, Once a year or less, Never), and how often they emailed in the past year (same answer choices). These questions are listed in the Appendix. We then created a 5 level frequency of internet use variable and a 4 level frequency of email use variable (0 = no access or no use in past year, 1 = once a year to several times a year, 2 = several times a month, 3 = several times a week, 4 = every day). The test-retest reliability for frequency of internet and email use from one annual assessment to the next was good (kappa = 0.65 and 0.74, respectively). The internet use variables from the first decision making assessment are used in this analysis.

### Decision making assessment

Decision making is the ability to process multiple competing alternatives and choose a favorable outcome. A modified, 12-item version of a previously established performance-based measure was used to measure decision making (Finucane et al., 2005; Finucane and Gullion, 2010) in two specific domains that are particularly salient to the health, independence, and quality of life of older persons: healthcare and finances. This version of the decision making assessment tool has been described in detail (James et al., 2012), but briefly, participants are provided tables with information about HMO plans for the healthcare module, and information about mutual funds for the financial module. See the Appendix for a brief description of the decision making assessment. The information presented in the tables was designed to simulate materials used in financial and healthcare settings in the real world. Participants were then asked questions of varying difficulty levels for each module (3 simple and 3 complex for each) that assess comprehension and integration of the information in the tables and ability to choose the optimal HMO or mutual fund. For example, one of the simple healthcare questions presents information on a number of characteristics (member satisfaction, preventive care strategies, access to specialists, customer service, and premium costs) for three HMO plans and asks participants to select the HMO that is not below average on two specific characteristics. A complex health question presents similar information about nine HMO plans and asks participants to select the HMO that is not below average on four characteristics. Similarly, one of the simple financial questions presents information on a number of characteristics (gross annual return, management fee, minimum investment, years of activity) for three mutual funds and asks participants to select the mutual fund that is not below average on two specific characteristics. A complex financial question presents similar information about nine mutual funds and asks participants to select the mutual fund that is not below average on four characteristics. The total decision making score is the number of items answered correctly (range = 0–12). The decision making score was approximately normally distributed (median = 8, mean = 7.6, *SD* = 2.8) with a negative skew (skewness = −0.8). In previous research, the decision making measure has been shown to have adequate psychometric properties including high inter-rater reliability and short-term temporal stability (Finucane et al., 2005; Finucane and Gullion, 2010). Internal consistency was adequate (standardized alpha = 0.78). The decision making cohort has been related to cognition (Boyle et al., 2012b), susceptibility to scams (James et al., 2013), and mortality (Boyle et al., 2013).

### Other covariates

Age (based on date of birth and date of decision making assessment), sex, and education (years of schooling) were self-reported. A summary index of global cognition was derived from the average of z-scores from a battery of 19 neuropsychological tests (immediate and delayed recall of story A from Logical Memory, immediate and delayed recall of the East Boston Story, Word List Memory, Word List Recall, Word List Recognition, Boston Naming Test, Verbal Fluency, a 15-item reading test, Digit Span Forward, Digit Span Backward, Digit Ordering, Symbol Digit Modalities Test, Number Comparison, 2 indices from a modified version of the Stroop Neuropsychological Screening Test, a 15-item version of Judgment of Line Orientation, and a 16-item version of Standard Progressive Matrices; Wilson et al., 2005).

SES—Income was measured using the show card methodology; participants were shown a card with the following 10 possible categories and asked to choose the level that represented their annual income: 1: $0–$4999, 2: $5000–$9999, 3: $10000–$14999, 4: $15000–$19999, 5: $20000–$24999, 6: $25000–$29999, 7: $30000–$34999, 8: $35000–$49999, 9: $50000–$74999, 10: >$75000 (Bennett et al., 2005).

Psychosocial—Depressive symptoms over the past week were measured with a 10-item version of the Center for Epidemiologic Studies Depression (CES-D10) Scale (Kohout et al., 1993; Bennett et al., 2005). Loneliness was measured with a modified version (Wilson et al., 2007a) of the Jong-Gierveld Loneliness Scale (de Jong-Gierveld, 1987); the score ranged from 1 to 5, with higher values indicating more loneliness. Social support was assessed with four questions from the Multidimensional Scale of Perceived Social Support (Zimet et al., 1988) that make up the Significant Other subscale (Zimet et al., 1990); the score ranged from 1 to 5, with higher scores denoting more social support. Social network size was the total number of children, other relatives, and close friends seen at least once per month (Bennett et al., 2006). Because of skew, the number of contacts was square-rooted for regression analysis.

Functional status—Chronic medical conditions were the sum of self-reported medical condition items (hypertension, diabetes, heart disease, cancer, thyroid disease, and head injury with loss of consciousness). Instrumental activities of daily living (IADLs) were assessed using items from the Duke Older Americans Resources and Services project (Lawton and Brody, 1969); participants rated their ability to perform (no help, help, unable to do) eight activities: telephone use, meal preparation, money management, medication management, light and heavy housekeeping, shopping, and local travel. Life space, the extent of movement through the environment during daily functioning, was measured by self-report: participants were asked whether or not they had been in 6 zones within their surrounding environment in the past week. The smallest categories were collapsed due to small cell sizes, with resulting life space scores ranging from homebound (score = 0) to out of town (score = 4).

Literacy—Literacy is the ability to understand and interpret information and written materials in specific contexts (Kutner et al., 2006), such as the ability to understand drug risk information in the health domain, and the ability to calculate interest rates in the financial domain. Health and financial literacy were assessed with a series of questions designed to measure knowledge of health and financial information and concepts, and numeracy as previously described (Lusardi and Mitchell, 2006; James et al., 2012). The entire health and financial literacy scale is available as an Appendix to a previous publication (James et al., 2012). The total literacy score was expressed as the percentage correct out of total items (from 0 to 100). Internal consistency was adequate (standardized alpha = 0.79).

### Statistical analysis

We first examined distributions of answers to our questions about internet access and frequency of usage, as well as correlations of these variables with covariates and with each other using Spearman correlation coefficients. We then used linear regression models to examine the association between internet access (access vs. no access), frequency of internet use (5 category scale), and frequency of email use (5 category scale) with financial and healthcare decision making. All models were adjusted for age, sex, race, education, and global cognition. We also constructed fully adjusted models that included these terms plus terms for SES, psychosocial factors, functional status, and literacy. We then ran the fully adjusted model with data from only the participants that reported access to the internet. Finally, we tested for effect modification of the association of frequency of internet use and financial and healthcare decision making by constructing a series of models with terms for internet use, the covariate (centered around its mean), and an interaction term for the covariate by internet use frequency; each interaction was examined separately. All parameter estimates are reported as standardized β coefficients, which represents the difference in standard deviations of the outcome score associated with a one standard deviation increase in the independent variable. Linear assumptions of regression models were tested by replacing ordinal categorical variables with dummy variables for each category. Model diagnostics were performed by checking residuals using analytic and graphical techniques. All analyses were conducted using SAS 9.3.

## Results

Seventy percent of participants reported that they had access to a computer with internet service. Men were more likely to report access to the internet than women (84.4 vs. 63.4% respectively, χ^2^ = 25.0, *p* < 0.001). Of those with internet access, 17.0% did not use the internet, 28.5% reported they used or searched the internet several times a year, 13.6% several times a month, 19.3% several times a week, and 35.7% used the internet every day. Of those with internet access, 21.2% did not use email, 9.3% reported they emailed several times a year, 10.8% emailed several times a month, 18.6% emailed several times a week, and 40.0% emailed every day. By combining persons with no internet access and persons who reported internet access but no internet use into one category, 58.2% of the sampled cohort reported some use of the internet (Table 1). Differences between those with and without internet access are reported in Table 2.

**Table 1:** **Frequency of internet use and email use**.

**(*n* = 661)**	**Internet use (%)**	**Email use (%)**
No use	276 (41.8)^*^	297 (44.9)^**^
Several times a year or less	68 (10.3)	43 (6.5)
Several times a month	63 (9.5)	50 (7.6)
Several times a week	89 (13.5)	86 (13.0)
Every day	165 (25.0)	185 (28.0)

* 77 people (27.9% of those who did not use the internet) had access to the internet but did not surf the internet.

** 98 people (33.0% of those who did not use email) had access to the internet but did not use email.

**Table 2:** **Characteristics of cohort by internet access, and correlations of internet use frequency with characteristics**.

**Characteristic**	**All participants (*n* = 661), mean (*SD*) or %**	**No internet access (*n* = 199), mean (*SD*) or %**	**With internet (*n* = 462), mean (*SD*) or %**	*p*-value for difference^*^	**Spearman correlation ρ with frequency of use in those with access (*p*-value)**
Age	82.2 (7.6)	85.1 (6.8)	80.7 (7.5)	<0.001	−0.32 (*p* < 0.001)
Female sex	76.3%	88.9%	70.8%	<0.001	0.04 (*p* = 0.38)
White, non-Hispanic	91.4%	92.0%	91.1%	0.73	−0.07 (*p* = 0.13)
Education	15.2 (3.0)	13.7 (2.6)	15.8 (3.0)	<0.001	0.20 (*p* < 0.001)
Income^**^	7.2 (2.4)	6.0 (2.5)	7.6 (2.2)	<0.001	0.12 (*p* = 0.01)
Global cognition	0.2 (0.5)	−0.1 (0.5)	0.4 (0.5)	<0.001	0.34 (*p* < 0.001)
Depressive symptoms (CESD-10)	1.0 (1.6)	1.5 (2.0)	0.7 (1.2)	<0.001	−0.06 (*p* = 0.20)
Loneliness	2.2 (0.6)	2.4 (0.6)	2.1 (0.6)	<0.001	−0.10 (*p* = 0.03)
Social networks^***^	2.4 (1.0)	2.1 (0.9)	2.5 (1.0)	<0.001	0.07 (*p* = 0.12)
Social support	4.4 (0.7)	4.3 (0.6)	4.4 (0.7)	0.007	0.12 (*p* < 0.001)
Chronic conditions	0.9 (1.0)	0.9 (1.0)	0.9 (1.0)	(0.58)	0.02 (*p* = 0.70)
IADL disability	1.3 (1.8)	2.1 (2.1)	0.9 (1.5)	<0.001	−0.24 (*p* < 0.001)
Life space	3.5 (1.0)	3.2 (1.2)	3.7 (0.8)	<0.001	0.20 (*p* < 0.001)
Literacy	0.7 (0.1)	0.6 (0.1)	0.7 (0.1)	<0.001	0.34 (*p* < 0.001)
Decision making	7.6 (2.8)	6.0 (3.0)	8.3 (2.5)	<0.001	0.35 (*p* < 0.001)

* From tests of differences in characteristics between those with and without internet access: t-test for continuous variables and chi-square for categorical variables.

** Income range: 1 (USD < 5,000) to 10 (USD > 75,000). A score of 7 represents an annual income of USD 30,000–34,999.

*** Number of children, other relatives, and close friends seen at least once per month, squared.

Higher frequency of internet use in those with internet access was correlated with lower age, higher education, higher income, higher cognitive function, less loneliness, more social support, less IADL disability, larger life space, and higher level of literacy (Table 2). There was no difference in frequency of internet use by race/ethnicity, depressive symptoms, social network size, or number of chronic medical conditions. Internet access was correlated with higher frequency of internet use (ρ = 0.70, *p* < 0.001) and higher frequency of email use (ρ = 0.67, *p* < 0.001); frequency of internet use was associated with frequency of email use (ρ = 0.84, *p* < 0.001).

### Internet access and decision making

In a linear regression model adjusted for age, sex, race, education, and global cognition, internet access (access vs. no access) was associated with better decision making (β = 0.09, *p* = 0.008). Because persons with low SES, certain psychosocial factors, poor functional status, and low health and financial literacy may be less likely to use the internet and also may have poorer decision making abilities, we further adjusted for these potential confounders. In a model further adjusting for income, depression, loneliness, social network size, social support, chronic conditions, IADL disability, life space, and literacy, the association between internet access and decision making was not statistically significant (β = −0.03, *p* = 0.36).

### Frequency of internet and email use and decision making

In linear regression models adjusted for age, sex, race, education, and global cognition, higher frequency of internet use (Table 3, Model 1) and higher frequency of email use (Table 3, model 2) were associated with better decision making. To put the internet use frequency finding in context, a person who used the internet every day displayed on average a 0.26 standard deviation increase in decision making ability as compared to a person who did not uses the internet. In models further adjusting for income, chronic conditions, depression, loneliness, social network size, social support, IADL disability, life space, and literacy, higher frequency of internet access was associated with decision making (Table 3, model 3), but higher frequency of email use was not (Table 3, model 4). We also examined the adjusted association of frequency of internet and email use in only those who reported having access to the internet and the finding was unchanged (internet frequency β = 0.091, *p* = 0.030; email frequency β = 0.049, *p* = 0.24).

**Table 3:** Associations of the frequency of internet and email use (independent variables) with financial and healthcare decision-making ability (dependent variable)^*^.

	**Model 1**		**Model 2**		**Model 3**		**Model 4**	
	**β**	***p*-value**	**β**	***p*-value**	**β**	***p*-value**	**β**	***p*-value**
Age	−0.17	<0.001	−0.18	<0.001	−0.10	0.004	−0.11	0.002
Male sex	0.15	<0.001	0.16	<0.001	0.08	0.015	0.09	0.007
Race (Not white, Hispanic)	−0.11	<0.001	−0.11	<0.001	−0.06	0.067	−0.06	0.091
Education	012	<0.001	0.12	<0.001	0.08	0.025	0.08	0.019
Global cognition	0.43	<0.001	0.44	<0.001	0.29	<0.001	0.29	<0.001
Income^**^					0.06	0.076	0.06	0.077
Depressive symptoms (CESD-10)					0.04	0.27	0.03	0.30
Loneliness					0.03	0.39	0.03	0.36
Social networks^***^					−0.02	0.56	−0.01	0.63
Social support					0.01	0.73	0.01	0.76
Chronic conditions					0.00	0.88	0.01	0.83
IADL disability					−0.03	0.39	−0.03	0.35
Life space					0.07	0.028	0.07	0.033
Literacy					0.28	<0.001	0.29	<0.001
Internet use frequency	0.11	0.002			0.08	0.024		
Email frequency			0.08	0.017			0.05	0.13
*N*	661		661		627		627	
*R*^2^	0.44		0.44		0.49		0.49	

* β = standardized Beta coefficient (change in decision making score associated with a one standard deviation increase in independent variable).

** Income range: 1 (USD < 5000) to 10 (USD > 75,000). A score of 7 represents an annual income of USD 30,000–34,999.

*** Number of children, other relatives, and close friends seen at least once per month, squared.

### Effect modification of the association of internet use frequency and decision making

**Figure 1:**
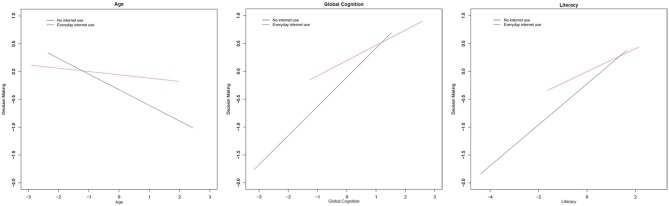
**Significant interactions of frequency of internet use with covariates on decision making ability**. All models included terms for age, sex, race, education, and global cognition. Based on standardized beta coefficients. Red line indicates regression line for persons who reported using the internet every day. Black line indicates the regression line for persons who reported no internet use. All interactions presented are statistically significant (*p* < 0.05).

We tested whether there was effect modification by internet use for all of the covariates examined in these analyses using interaction models that included terms for the cross-product of internet use frequency and covariates. Significant interactions are summarized in the Figure 1. There was evidence of interaction by age, such that on average, the negative relationship between older age and decision making ability was attenuated in persons that used the internet frequently (Table 4, *Age interaction*). There was also evidence of interaction by cognition, indicating that the relationship between cognitive function and decision making ability was not as strong in persons who use the internet more frequently (Table 4, *Cognition interaction*). Finally, there was evidence of interaction by literacy, such that the association between level of literacy and decision making ability was attenuated in those who used the internet more frequently (Table 4, *Literacy interaction*).

**Table 4:** Significant interactions of internet use frequency with covariates on healthcare and financial decision making (dependent variable)^*^.

	**Age interaction**		**Cognition interaction**		**Literacy interaction**	
	**β**	***p*-value**	**β**	***p*-value**	**β**	***p*-value**
Internet use frequency	0.10	0.005	0.12	<0.001	0.09	0.008
Age	−0.28	<0.001				
Age × internet use frequency	0.13	0.003				
Cognition			0.52	<0.001		
Cognition × internet use frequency			−0.14	0.001		
Literacy					0.36	<0.001
Literacy × internet use frequency					−0.09	0.029
*N*	661		661		661	
*R*^2^	0.45		0.45		0.49	

* β = standardized Beta coefficient (change in decision making score associated with a one standard deviation increase in independent variable). All models included terms for age, sex, education, race, and global cognition; only terms for variables in interaction tested are reported.

## Discussion

We found that over two-thirds of a cohort of almost 700 community-dwelling older adults reported access to the internet, and of those with internet access, over half reported both searching the internet and using email at least several times a week. A higher frequency of internet use was associated with better decision making ability regarding health and financial choices, even after adjusting for a number of factors that could potentially account for the association. There was evidence that the association was stronger for certain older adults who may be most at risk for bad decision making, specifically those who were oldest, had the lowest levels of cognition, were homebound or did not travel far from home, and were the least literate. This research supports the hypothesis that use of the internet is associated with better financial and healthcare decision making among older persons. Further, this association may be strongest in those who are at highest risk of poor decision making.

This study supports the findings from a 2012 Pew report indicating that over half of persons over age 65 are actively online (Zickuhr and Madden, 2012). Though internet use has been associated with positive psychosocial outcomes in previous research on older adults (Chen and Persson, 2002; Mcmellon and Schiffman, 2002; Karavidas et al., 2005; Shapira et al., 2007), there is little data on the relationship of internet and decision making in seniors. In secondary qualitative interviews with older adults participating in a computer intervention trial, participants exposed to an internet training program expressed the belief that the activities contributed to their decision making and critical thinking, though this was not empirically tested (Shapira et al., 2007). Another computer training intervention trial found that while older adults were willing to use the internet as a source for general health information, internet training courses did not alter healthcare decision making patterns—instead participants adhered to a physician-centered model of care (Campbell and Nolfi, 2005). Our study was one of the first to examine differences in decision making ability based on frequency of internet use in the daily lives of older persons in the community. Because it was an observational study, it was important to adjust for variables that could confound the relationship between internet use and financial and healthcare decision making. We found that the association held despite adjustment for differences in demographics, cognitive abilities, SES, psychosocial factors, functional status, and literacy with health and financial concepts.

The mechanisms linking internet use to better financial and healthcare decision making in late life are unknown. Interestingly, while the association between frequency of internet use and financial and healthcare decision making was robust to adjustment for confounders, the association for simply having access to the internet and financial and healthcare decision making was not statistically significant, though there was a trend. This may suggest that actual use of the internet rather than simply having access to it (or the characteristics that determine who has access) is driving the association. This is in line with the re-characterization of the Digital Divide by certain researchers as a Digital Spectrum, in which the most significant differences may not be found between users and non-users but between those who do and do not use the internet frequently, efficiently, and effectively (Hargittai, 2002; Lenhart and Horrigan, 2003; Freese et al., 2006). Further, email use, specifically, was not significantly associated with financial and healthcare decision making after adjustment for confounders. This argues against the notion that increased access to existing social networks through email communication is responsible for better financial and healthcare decision making (Boase et al., 2006). The finding that more frequent email use is not associated with better financial and healthcare decision making while more frequent internet use in general is may suggest that surfing the internet can improve access to information needed for effective decision making, though we cannot know for sure this is the case given the study design (Jadad et al., 2000). Older adults typically display low levels of health and financial literacy necessary for making informed decisions (James et al., 2012) and often cannot easily travel from their home to gather information due to functional limitations. Thus, the internet may more readily bring the resources required for informed decision making about finances and healthcare into an older person's purview. Alternatively, surfing the web may have a direct neurological effect on the brain. Recent neuroimaging research has shown that internet use leads to increased activation in areas of the brain related to decision making (Small et al., 2009). Surfing the web may constitute a form of mentally stimulating activity, which has been linked to preserved cognitive performance in later life (Wilson et al., 2007b, 2012). However, this cross-sectional observational study cannot establish that internet use leads to better financial and healthcare decision making, and we cannot rule out the alternate hypothesis that better decision makers are more likely to use the internet (reverse causation), or that some common third factor (such as personality) leads to both higher internet use and better performance on the decision making assessment (unmeasured confounding).

The results of this study have positive implications for the health, financial security, and independence of older persons for three important reasons. First, older Americans face a complex array of health and financial choices within a context of increased physical limitations and health burdens, lack of new revenue streams, and limited opportunity and time to recover from bad decisions. Older adults often must follow complicated prescription regimens for multiple medications, make choices on the timing of transitions to assisted living or nursing home, and make preparations for end-of-life medical care. Financially, they must decide how to live off of fixed incomes such as social security and retirement savings, intergenerational transfers of wealth, while simultaneously being at high risk of being financially victimized by family, friends, or strangers (Metlife Inc, 2011). Second, while faced with these complicated and important decisions, older persons are highly vulnerable to poor decision making (Finucane et al., 2002; Denburg et al., 2005; Boyle et al., 2012b). Increasing age is associated with greater comprehension errors and inconsistent preferences (Finucane et al., 2002), and older adults are much more likely to passively defer to physicians or companions in medical decisions (Beisecker, 1988) and to be fooled into financial victimization (AARP, 1996). Third, the internet is quickly becoming a primary decision making tool for most Americans. A 2005 Pew survey found that nearly a third (29%) of Americans said the internet played a crucial role in at least one major decision in the previous year (Boase et al., 2006). Given the Digital Divide between young and old in internet use (Loges and Jung, 2001; van Dijk and Hacker, 2003; Zickuhr and Madden, 2012), fewer older Americans are taking advantage of the internet as a potential decision making aide. Moreover, the internet is becoming increasingly “proto-normative” as more and more services and information sources are being moved online with the assumption that those who require these resources are efficient internet users (Freese et al., 2006). An example is the online tool provided by the United States government to help seniors navigate the prescription drug benefit for seniors, Medicare Part D (Centers for Medicare, and Medicaid Services). In sum, the age group facing many of the most complex and significant life decisions may paradoxically be the least likely to reap the benefits of modern society's burgeoning technological revolution (Hart et al., 2008). This study provides initial but important evidence that seniors who do plug into the digital world display better decision making capabilities, though more research is required to understand whether this indicates that more internet use can improve financial and healthcare decision making.

Importantly, we found evidence that more frequent internet use may attenuate the negative associations of other predictors of poor financial and healthcare decision making. Results of interaction models indicated that while older age, poorer cognitive function, and lower levels of health and financial literacy were associated with inferior healthcare and financial decision making, a higher frequency of internet use attenuated these associations. This suggests that the internet may provide a decision making aide for vulnerable groups of older adults such as those with mild cognitive impairments that may diminish their decision making abilities, and those who possess less knowledge of health and financial concepts, though future research is necessary to support such a claim.

There are limitations of this study. First, the cross-sectional, observational design of this study prevents causal inferences; that is, we cannot determine whether internet use influences decision making abilities or vice versa. This study establishes an association only, not a causal role for internet use. Second, the older adults comprising this volunteer cohort are predominantly white, highly educated, and live in retirement communities (some of which have computer rooms available) in or around a major metropolitan area. Therefore, these results—especially in regards to access to and frequency of internet use—may not generalize to the older population in general; in particular, persons who are more highly educated have more access to the internet and display better decision making ability in general, so we may be observing only the upper range of this association. Assessment of internet use was based on self-report, and therefore may not accurately reflect the true patterns of internet use in this group. Although the decision making assessment was based on decisions faced in the real world by older adults, we were not able to assess actual health and financial decisions made by participants in their lives. Another limitation was the inability to control for offline computer usage, so we are unable to say for certain whether internet use specifically or computer use in general drives the association with financial and healthcare decision making. However, we believe that internet use drives acquisition of financial and health knowledge and new learning, and it is not clear how offline usage would have the same effect. This study had a number of strengths including a detailed assessment of decision making ability in a fairly large cohort of community-dwelling older adults who did not have dementia, and the ability to adjust for a large number of variables that could potentially confound this relationship including a robust measure of cognitive ability and a measures of SES, psychosocial factors, functional status, and literacy, though unmeasured confounding could still be present. This study is one of the first that we are aware of to provide evidence supporting a relationship between internet use and heightened decision making abilities in older adults, yet future research, including experiments with internet interventions, is needed to establish whether increasing internet use can improve decision making ability. Future work in our cohort will include longitudinal analyses to determine whether frequency of internet use influences age-related or disease-related changes in decision making abilities.

### Conflict of interest statement

The authors declare that the research was conducted in the absence of any commercial or financial relationships that could be construed as a potential conflict of interest.

## Appendix

### Internet use assessment

1. Question: Do you have access to a computer with internet service?
  1 = Yes
  2 = No
2. Question: During the past year, how often did you use or search the internet?
  1 = Every day or almost every day
  2 = Several times a week
  3 = Several times a month
  4 = Several times a year
  5 = Once a year or less
  6 = Never
3. Question: During the past year, how often did you email?
  1 = Every day or almost every day
  2 = Several times a week
  3 = Several times a month
  4 = Several times a year
  5 = Once a year or less
  6 = Never

(“Once a year or less” and “several times a year” categories combined in analyses due to small cell size).

### Decision making assessment

Items C-1a, C-1b, C-2, C-3a, C-3b, C-4, (healthcare) and C-9a, C-9b, C-10, C-11a, C-11b, C-12 (financial) from the original Decision Making Competence Assessment tool were used in this analysis, with slight modifications to the presentation of the information to participants in order to make it more accessible to older persons (larger font; empty circles, half circles, and full circles replaced with −, +, and ++, respectively; third-person names such as “David,” “Susan,” and “Joe” replaced with the second-person: “you”). The original tool is available online as an Appendix to Finucane et al. (2005): http://supp.apa.org/psycarticles/supplemental/pag_20_1_71/PAG.Finucane0128.sup.pdf
